# Postpartum-acquired hemophilia a: case report

**DOI:** 10.17843/rpmesp.2023.402.12593

**Published:** 2023-06-30

**Authors:** Carlos H. Contreras-Pizarro, Gloria Chumpitaz-Anchiraico, Rosario Ticona Sanjínez, Carlos Peña-Diaz

**Affiliations:** 1 Faculty of Medicine, Universidad Nacional Mayor de San Marcos, Lima, Peru. Universidad Nacional Mayor de San Marcos Faculty of Medicine Universidad Nacional Mayor de San Marcos Lima Peru; 2 Hospital Nacional Edgardo Rebagliati Martins, EsSalud, Lima, Peru. Hospital Nacional Edgardo Rebagliati Martins EsSalud Lima Peru

**Keywords:** Hemophilia A, Postpartum Period, Back Pain, Hematuria

## Abstract

Acquired hemophilia A is a rare bleeding disorder worldwide, characterized by the presence of inhibitory autoantibodies directed against a coagulation factor, most often factor VIII. There are several possible causes, and it can occur during the postpartum period. We present the case of a 34-year-old female patient with back pain, hematuria and a right gluteal hematoma, with no previous history of bleeding. She was transferred to the emergency department due to the extension of the hemorrhagic manifestations. Diagnosis was confirmed with the coagulation profile, mixing test and the assessment of factor VIII inhibitor tier. The case highlights the importance of considering this condition in a postpartum patient with persistent postoperative bleeding, extensive hematoma and no history of previous bleeding.

## INTRODUCTION

Acquired hemophilia A (AHA) is caused by the presence of antibodies directed against coagulation factors, the most common being factor VIII ^1^. AHA is an uncommon condition and its incidence (estimated at 1.5 cases per million persons/year) ^2^ may be underestimated due to lack of knowledge, registry limitations, or a fulminant clinical presentation, which hinders confirmation of the diagnosis ^1^.

The age distribution of AHA is usually biphasic, with a first peak between 20 and 30 years of age, mainly in young women during pregnancy and postpartum, and a second peak in those over 60 years of age, with no gender differences ^3^. The mortality rate is 41% if patients do not receive treatment within the first week ^4^.

The cause of AHA is unknown in 50% of the cases; of the remaining cases, 7-21% are related to the postpartum period ^5^. The mortality rate of postpartum-acquired hemophilia A ranges from 12 to 22% and it may occur in pregnant women who present bleeding without apparent cause and who have no history of bleeding disorders ^4^. It usually occurs after the first pregnancy in 80% of cases, and generally one to four months after delivery ^6^^,^^7^.

The Hemophilia Unit of the Hematology Department of the Edgardo Rebagliatti Martins National Hospital (HNERM) of the Social Health Insurance (EsSalud) is one of the most important hemophilia centers in Peru ^8^. Although there is information on the prevalence of congenital hemophilia, there is no institutional registry of the epidemiological characteristics, diagnostic criteria and treatment that these patients receive ^9^; in addition, few cases have been reported ^9^^,^^10^, which is why it is important to notify them in order to ensure the timely recognition of this disease.

We present the case of a patient with lumbar pain, hematuria, and a hematoma in the right gluteal region two months after her second pregnancy. Due to the increasing hemorrhagic manifestations and alteration of vital signs, she was transferred to the emergency department of the HNERM, where postpartum AHA was confirmed. 

## CASE REPORT

The patient was a 34-year-old female with a four-week illness. Two months earlier, she delivered at 37 weeks by cesarean section and had persistent bleeding from the operative wound. She denied any history of bleeding during childhood or adolescence. Three years earlier, she gave birth to her first child (also by cesarean section), who died due to a chromosomopathy (according to the patient). In addition, she stated to be allergic to tramadol.

Lumbar pain due to bilateral renal lithiasis was the first symptom. Subsequently, she expelled a kidney stone and presented hematuria for three days, for which she received tranexamic acid every 12 hours. Three weeks later, she experienced pain in the lower region of the left thigh that increased in intensity, with stiffening of the area. Due to persistent symptoms, she was prescribed intramuscular diclofenac, which caused ecchymosis and bleeding in the buttock and persisted despite compression with gauze.

A doppler ultrasound revealed deep venous thrombosis of the left lower limb; she attended the local hospital with these results. She received subcutaneous enoxaparin 30mg/24hrs, in addition to morphine, and was hospitalized. The following day, she presented epigastric pain, blurred vision, heart rate of 117 beats/min, blood pressure of 113/85 mmHg and oxygen saturation of 93%. Treatment with enoxaparin stopped. Blood tests revealed a hemoglobin of 6.4 g/dL, which represented a difference of 4 g/dL with respect to the result from one day before admission, which was 10.4 g/dL. Due to the above, she received two blood units. Vasculitis was considered as a possible diagnosis, so she was prescribed methylprednisolone and was referred to the HNERM.

**Figure 1:**
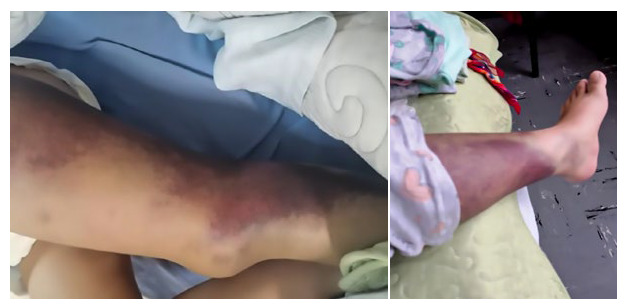
Ecchymotic lesions in a patient with postpartum-acquired hemophilia A.

Physical examination on admission revealed severe pallor, extensive ecchymosis on the left thigh and lateral aspect of the left knee, as well as a hematoma on the right thigh (Figure 1). The hemogram showed normocytic and normochromic moderate anemia (Hb=9.8 g/dL). Her blood glucose level was at 160mg/dl. The values of AST and ALT were 52 U/L and 86 U/L, respectively. The coagulation profile showed prolongation of the activated partial thromboplastin time (aPTT) of 91.2 seconds. The rest of the hemogram, biochemistry, electrolytes, liver profile and coagulation profile were normal. Soft tissue ultrasound of the right buttock revealed a collection at the level of the subcutaneous cellular tissue (SCCT) and edema up to the upper third of the thigh. Doppler ultrasound of the left lower limb showed adequate flowmetry with no signs of thrombosis in the common, superficial and deep femoral vein.

She received symptomatic treatment; blood and urine cultures were negative. The values of antinuclear antibodies (ANA), complement C3 and C4 and ferritin were within the normal range.

The mixing test was used to confirm the hemophilia diagnosis, and it showed a partial correction of aPTT. Factor VIII was low (<1.0 U/dl), and we found the factor VIII inhibitor (8.64 Bethesda units/ml). Table 1 describes the evolution of the laboratory tests. The tests confirmed this was a case of AHA, which was considered to be related to the postpartum period due to the onset of symptoms.

**Table 1:** Evolution of coagulation profile, factor dosage and factor VIII inhibitor in a patient with postpartum-acquired hemophilia.

	Admission	Remission		Reference
		Day 21	Day 47	
Coagulation profile				
aPTT ^a^	71.7	46.8	28.93	25-37 seg
Prothrombin activity %	130.9	140.4	123.8	80-120%
Fibrinogen	270.16	111.42	221.46	200-400 mg/dl
Coagulation factors				
F VIII	<1.0	1.58	42.7	50-150 U/dl
F IX	224.2	No ^b^	No b	50-150 U/dl
F XI	123.8	No b	No b	50-150% U/dl
Factor VIII inhibitor				
Titer	8.64	3.92	<0.6	<0.6 UB/ml

a Activated partial thromboplastin time (aPTT).

b Subsequent measurements were not necessary because values on admission were normal.

She started receiving prednisone 50mg PO at breakfast and 10mg PO at lunch, cyclophosphamide 50mg 2 tablets PO every 24hrs as well as hemophilic anti-inhibitor coagulant complex (FEIBA). Five days later, the latter drug was discontinued due to oppressive chest pain, dyspnea and nausea (possible adverse drug reaction), so she started receiving recombinant activated factor VII (NovoSeven).

The patient progressed favorably, with a decrease in ecchymosis and without other symptoms, so she was discharged from the hospital.

## DISCUSSION

We present the case of a patient with postpartum AHA, two months after her second pregnancy, who progressed favorably after treatment with activated recombinant factor VII. The prevalence rate of this condition is 8.4% of all cases of acquired hemophilia ^4^. Cases of postpartum AHA after the second pregnancy are rare, since 80% of the cases occur after the first pregnancy ^4^. A systematic review showed that this condition can occur up to the seventh pregnancy ^11^.

The etiopathogenesis of AHA involves a combination of genetic, immune and environmental factors ^12^^,^^13^. A significantly increased frequency of human leukocyte antigen (HLA) class II alleles (HLA-DRB1*16 and HLA- DQB1*0502) ^12^ has been detected in AHA cases; in addition to single nucleotide polymorphisms of the CTLA-4 gene ^14^, leading to the absence of CTLA-4-dependent regulatory mechanisms of T lymphocyte functions.

It is important to bear in mind that the factor VIII inhibitor can be transferred through the placenta from the mother to the fetus. Therefore, the newborn may also be affected ^11^, causing complications such as intracranial hemorrhage, severe epistaxis and hematemesis^11^^,^^15^. A postnatal cranial ultrasound should be performed before hospital discharge in the rare cases of peripartum AHA ^(^^16^.

Bleeding usually occurs in the skin and soft tissues in cases of acquired hemophilia ^17^^,^^18^ (Figure 1). Hemarthrosis is present in 4.9% of patients ^6^ and is more frequent in congenital hemophilia. Hematuria is the second most common symptom and is accompanied by other hemorrhagic manifestations such as ecchymosis or hematomas (as in the present case). Isolated hematuria is extremely rare ^19^. Diagnosis is usually made at a median of 60 days after delivery, with a range of 0 to 308 days ^9^.

Enoxaparin worsened the patient’s symptoms. In this regard, the risk of uncontrolled bleeding almost always outweighs the benefit of anticoagulation in patients with bleeding disorders, hereditary or acquired ^20^. In a review of nine cases of patients with acquired hemophilia A and recurrent thrombosis, the authors prioritized the treatment of bleeding and discontinued anticoagulation at the onset of bleeding manifestations ^21^. Since the patient already presented hemorrhagic manifestations, greater caution should have been exercised in the administration of this drug.

Postpartum AHA diagnosis is based on the increase of aPTT time associated with hemorrhagic manifestations in patients with no previous family or personal history of coagulopathy ^22^. It is important to rule out other congenital coagulopathies, the presence of lupus anticoagulant and disseminated intravascular coagulation ^22^, as in the present case.

The treatment of AHA includes two treatment lines: 1) the use of drugs for the control of active bleeding and 2) the eradication of factor inhibitors by immunosuppressants ^4^. The first line includes bypassing agents (recombinant factor VIIa or activated prothrombin complex concentrate, commercially known as FEIBA) and recombinant porcine factor VIII. The patient in our case had adverse reactions to FEIBA (oppressive chest pain, dyspnea and nausea). In this regard, current literature recommends not to exceed 200 units/kg/24hrs of FEIBA, because it may be associated with a risk of venous thromboembolism or disseminated intravascular coagulation ^23^^,^^24^, and it should not be administered together with tranexamic acid, since this is associated with thromboembolic events ^23^^,^^24^. The second treatment line starts with a combination of steroids alone (dose of 1mg/kg/day) or associated with cyclophosphamide at low doses (1-2 mg/kg/day), for 3 to 5 weeks ^25^. In the event of failure of first-line treatment, the alternative of choice is rituximab ^(^^25^. The usual recommendation consists of weekly doses of 375 mg/week for 4 weeks ^26^^,^^27^.

Partial correction when performing the mixing test or correction with normal plasma has also been mentioned in previous reports ^15^. This could be due to analytical and pre-analytical aspects that are beyond the scope of this report ^27^. The detailed anamnesis, added to the laboratory findings (reduction of factor VIII activity, presence of anti-FVIII inhibitors 0.6 Bethesda Units/ml) ^28^, and the response to treatment allowed establishing the final diagnosis.

One of the limitations of this report is the fact that enoxaparin was administered to a patient with an acquired coagulation disorder, due to the finding of deep venous thrombosis in the left lower limb. This decision aggravated the patient’s condition. This finding could have been misinterpreted (the specificity of color Doppler ultrasound for diagnosis is operator-dependent, the literature mentions values between 62 to 83%) ^29^; and the second is that there was a thrombotic picture. However, in the latter situation, the hemorrhagic manifestations that the patient showed should have been considered, so she should have been referred in a timely manner for complementary examinations.

In conclusion, this report provides information on the diagnosis and treatment of a case of postpartum AHA. It is important to consider this condition in a postpartum patient with persistent bleeding from the operative wound, extensive ecchymosis and no history of bleeding.
