# Supplementary material for: Postpartum-acquired hemophilia a: case report
Source: Rev Peru Med Exp Salud Publica. 2023 Jun 30;40(2):242–6. doi: 10.17843/rpmesp.2023.402.12593 (PMC10953653; doi:10.17843/rpmesp.2023.402.12593)
Supplement: Translation

# Translation: Hemofilia A adquirida en el posparto: reporte de caso

**Authors:** Carlos H. Contreras-Pizarro, Gloria Chumpitaz-Anchiraico, Rosario Ticona Sanjínez, Carlos Peña-Diaz

**Affiliations:** 1 Facultad de Medicina, Universidad Nacional Mayor de San Marcos, Lima, Perú.; 2 Hospital Nacional Edgardo Rebagliati Martins, EsSalud, Lima, Perú.

**Keywords:** Hemofilia A, Periodo Posparto, Dolor de la Región Lumbar, Hematuria

## Abstract

La hemofilia A adquirida es un trastorno hemorrágico poco frecuente a nivel mundial, y se caracteriza por la presencia de autoanticuerpos inhibidores dirigidos hacia un factor de la coagulación, en la mayoría de ocasiones el factor VIII. Las etiologías son variadas, entre las que se encuentra el posparto. Se presenta el caso de una paciente de 34 años con dolor lumbar, hematuria y hematoma en región glútea derecha, sin antecedentes previos de sangrado. Por extensión de las manifestaciones hemorrágicas es transferida al servicio de emergencia. Los exámenes auxiliares de perfil de coagulación, prueba de mezclas y medición de los títulos de inhibidores del factor VIII permitieron confirmar el diagnóstico. El caso resalta la importancia de considerar esta patología en una paciente puérpera con persistencia de sangrado por herida operatoria, hematoma extenso y sin historia de sangrado previo.

## INTRODUCCIÓN

La hemofilia A adquirida (HAA) es una enfermedad que se produce por la presencia de anticuerpos que se dirigen contra los factores de la coagulación, siendo el más común el factor VIII ^1^. Se trata de una enfermedad infrecuente cuya incidencia (estimada en 1,5 casos por millón de personas/año) ^2^ puede estar infravalorada debido al desconocimiento de la misma, limitaciones de los registros, o una presentación clínica fulminante que impide su confirmación ^1^.

Se considera que la edad de presentación de la enfermedad es bifásica, con un primer pico entre los 20 a 30 años principalmente en mujeres jóvenes durante el embarazo y posparto, mientras que el segundo pico ocurre en mayores a 60 años, sin diferencias de sexo ^3^. La tasa de mortalidad es del 41% si los pacientes no reciben tratamiento dentro de la primera semana de presentación ^4^.

En relación a la etiología, son idiopáticas en el 50% de los casos, es decir, se desconoce la condición subyacente. Del porcentaje restante, el 7-21% de casos se relaciona con el posparto ^5^. En estos casos, la mortalidad varía entre 12 y 22%, y debe sospecharse en puérperas o gestantes que presentan hemorragia sin causa aparente y no tienen antecedentes de trastornos hemorrágicos ^4^. En el 80% de casos, suele aparecer luego del primer embarazo, y generalmente de uno a cuatro meses después del parto ^6^^,^^7^.

La Unidad de Hemofilia del Departamento de Hematología del Hospital Nacional Edgardo Rebagliatti Martins (HNERM) del Seguro Social de Salud (EsSalud), es uno de los centros de hemofilia más importantes del Perú ^8^. Si bien, se tiene información sobre las prevalencias de hemofilias congénitas, no se tiene un registro institucional de las características epidemiológicas, criterios diagnósticos y planes de tratamiento en estos pacientes ^9^, siendo escasos los reportes ^9^^,^^10^, por lo que resulta importante su comunicación para el reconocimiento oportuno de esta enfermedad.

Se presenta el caso de una paciente que dos meses después de su segundo embarazo presentó un cuadro de dolor lumbar, hematuria y hematoma en la región glútea derecha. Tras la extensión de manifestaciones hemorrágicas y alteración de las funciones vitales es transferida al servicio de emergencia del HNERM, donde se le se confirmó el diagnóstico de HAA en el posparto.

## REPORTE DE CASO

Se trata de una paciente de 34 años con un tiempo de enfermedad de cuatro semanas. Dos meses antes, había tenido un parto por cesárea en la semana 37 de embarazo, y presentaba sangrado persistente por la herida operatoria. Entre los antecedentes personales, negó haber presentado historia de sangrados durante la niñez o adolescencia. Tres años antes, había dado a luz a su primer hijo (parto también por cesárea), que falleció debido a una cromosomopatía (referida por la paciente). Además, manifestó ser alérgica al tramadol.

El cuadro clínico inició con dolor lumbar por litiasis renal bilateral. Posteriormente, logra expulsar un cálculo y luego de ello presentó hematuria por tres días, por lo que recibió ácido tranexámico c/12 h. Tres semanas después, presenta dolor en la región inferior del muslo izquierdo que fue incrementándose en intensidad, con endurecimiento de la zona. Por persistencia de los síntomas, se le indicó diclofenaco por vía intramuscular, que le provocó equimosis y sangrado en la zona del glúteo y que persiste pese a la compresión con gasas.

La paciente se realiza de manera particular una ecografía Doppler que revela trombosis venosa profunda de miembro inferior izquierdo, y acude al hospital de su localidad con dichos resultados. Se le administra anticoagulación con enoxaparina 30 mg/24 h subcutáneo, además de morfina para el manejo del dolor y es hospitalizada. Al día siguiente, presenta epigastralgia, visión borrosa, frecuencia cardiaca de 117 lat/min, presión arterial de 113/85 mmHg y saturación de 93%. Se decidió suspender la enoxaparina. El hemograma reveló una hemoglobina de 6,4 g/dl, que representaba una diferencia de 4 g/dl respecto al resultado un día antes de su ingreso, que fue de 10,4 g/dl. Debido a lo anterior, se le transfundió dos paquetes globulares. Ante la sospecha de vasculitis, se le indicó metilprednisolona y fue referida a nuestro hospital para ampliar el estudio.

En la exploración física al ingreso, se le encontró palidez severa, extensa equimosis en muslo y cara lateral de rodilla izquierda; además de un hematoma en muslo derecho (Figura 1). El hemograma reveló una anemia moderada (Hb=9,8 g/dl), normocítica y normocrómica. El examen bioquímico evidenció valores de glucosa de 160 mg/dl. Las enzimas hepáticas AST y ALT se encontraban en valores de 52 U/L y 86 U/L, respectivamente. El perfil de coagulación mostró prolongación del tiempo de tromboplastina parcial activada (TTPa) de 91,2 s. El resto de componentes del hemograma, bioquímico, electrolitos, perfil hepático y perfil de coagulación se encontraban en valores normales. La ecografía de partes blandas de la región glútea derecha reveló una colección a nivel del tejido celular subcutáneo (TCSC) y edema hasta el tercio superior del muslo. La ecografía Doppler en miembro inferior izquierdo mostró una adecuada flujometría sin signos de trombosis en vena femoral común, superficial y profunda.

**Figura 1:** Lesiones equimóticas en una paciente con hemofilia A adquirida en el posparto.

Se inició tratamiento sintomático y se solicitó hemocultivo y urocultivo, que resultaron negativos. Los valores de anticuerpos antinucleares (ANA), complemento C3 y C4 y ferritina estaban dentro del intervalo de referencia.

Ante la sospecha de hemofilia adquirida se solicitaron estudios para su confirmación, donde se encontró una corrección parcial de TTPa en la prueba de mezcla. Se midió el factor VIII encontrándose disminuida su actividad (<1,0 U/dl) y se demostró la presencia de un inhibidor del factor VIII: 8,64 unidades Bethesda/ml. En la Tabla 1 se describe la evolución de los resultados laboratoriales. Lo anterior permitió confirmar el diagnóstico de HAA, que se relacionó con el posparto por el inicio de los síntomas.

**Tabla 1:** Evolución del perfil de coagulación, dosaje de factores e inhibidor de factor VIII en una paciente con hemofilia adquirida en el posparto.

	Ingreso	Remisión		Valor de referencia
		Día 21	Día 47	
Perfil de coagulación				
TTPa ^a^	71,7	46,8	28,93	25-37 seg
% Actividad de protrombina	130,9	140,4	123,8	80-120%
Fibrinógeno	270,16	111,42	221,46	200-400 mg/dl
Factores de coagulación				
F VIII	<1,0	1,58	42,7	50-150 U/dl
F IX	224,2	No ^b^	No ^b^	50-150 U/dl
F XI	123,8	No ^b^	No ^b^	50-150% U/dl
Inhibidor de factor VIII				
Titulación	8,64	3,92	<0,6	<0,6 UB/ml

a Tiempo de tromboplastina parcial activada

b No fue necesaria su medición posterior debido a que resultaron normales al ingreso.

Se inició terapia con prednisona 50 mg VO en el desayuno y 10 mg VO en el almuerzo, ciclofosfamida 50 mg 2 tabletas VO c/24 h y complejo coagulante anti-inhibidor hemofílico (FEIBA). Cinco días después, se suspende el empleo de este último medicamento debido a dolor torácico opresivo, disnea y náuseas (posible reacción adversa medicamentosa), por lo cual se cambió a factor VII recombinante activado (NovoSeven).

La evolución clínica de la paciente fue favorable, con disminución de la equimosis y sin la presencia de otros síntomas, por lo que se decidió su alta hospitalaria.

## DISCUSIÓN

Se presenta el caso de una paciente con HAA en el posparto, dos meses después de su segundo embarazo, con evolución clínica favorable tras el tratamiento con factor VII recombinante activado. La prevalencia de esta patología es del 8,4% respecto al total de casos de hemofilia adquirida ^4^. Asimismo, el que se haya presentado luego del segundo parto es infrecuente, debido a que las estadísticas indican que el 80% de presentaciones ocurre en primigestas ^4^. Una revisión sistemática indica que esta patología puede presentarse hasta en el séptimo embarazo ^11^.

La etiopatogenia de la HAA involucra una combinación de factores genéticos, inmunes y medioambientales ^12^^,^^13^. Se ha detectado una frecuencia significativamente mayor de los alelos del antígeno leucocitario humano (HLA) de clase II (HLA-DRB1*16 y HLA- DQB1*0502) ^12^ en la HA; además de polimorfismos de un solo nucleótido del gen CTLA-4 ^14^, llevando a la ausencia de mecanismos reguladores de las funciones del linfocito T dependientes del CTLA-4.

Es importante tener en cuenta que el inhibidor del factor VIII puede transferirse a través de la placenta de la madre al feto. Por lo tanto, el recién nacido también puede verse afectado ^11^, generando complicaciones como hemorragia intracraneal, epistaxis severa y hematemesis ^11^^,^^15^. Se recomienda que en los raros casos de HAA periparto, se realice una ecografía craneal posnatal para cada bebé antes del alta hospitalaria ^16^.

Las zonas de sangrado en la hemofilia adquirida se suelen localizar en piel y tejidos blandos ^17^^,^^18^ (Figura 1); a diferencia de la hemartrosis, que está presente en el 4,9% de los pacientes ^6^ y es más frecuente en las hemofilias congénitas. La hematuria, ocupa el segundo lugar en frecuencia de presentación de los síntomas, y va acompañado de otras manifestaciones hemorrágicas como equimosis o hematomas (como en el presente caso). La hematuria aislada es extremadamente rara ^19^. El diagnóstico suele realizarse con una mediana de 60 días después del parto, con un rango de 0 hasta 308 días ^9^.

Se observa que la administración de enoxaparina empeoró los síntomas de la paciente. Al respecto, el riesgo de sangrado no controlado supera casi siempre el beneficio de la anticoagulación en pacientes con trastornos hemorrágicos, hereditarios o adquiridos ^20^. En una revisión de nueve casos de pacientes con hemofilia A adquirida y trombosis recurrente, todos los autores priorizaron el tratamiento del sangrado y suspendieron la anticoagulación ante la aparición de manifestaciones hemorrágicas ^21^. Debido a que la paciente ya presentaba manifestaciones hemorrágicas, se debió tener mayor precaución en la administración de este medicamento.

El diagnóstico de la HAA en el posparto se basa en un elevado nivel de sospecha ante la aparición de un alargamiento del tiempo de TTPa, asociado a manifestaciones hemorrágicas en pacientes sin historia familiar previa o personal de coagulopatía ^22^. Es importante descartar la presencia de otras coagulopatías congénitas, la presencia de anticoagulante lúpico y coagulación intravascular diseminada ^22^, como se realizó en el presente caso.

El tratamiento de la HAA consiste en dos líneas: 1) empleo de medicamentos para el control del sangrado activo y 2) erradicación de los inhibidores del factor mediante inmunosupresores ^4^. En el primer grupo, se incluye a los agentes de derivación (factor VIIa recombinante o concentrado de complejo de protrombina activado, conocido comercialmente como FEIBA) y factor VIII porcino recombinante. La paciente del caso presentó reacciones adversas hacia el FEIBA (dolor torácico opresivo, disnea y náuseas). Al respecto, la literatura recomienda no exceder de 200 unidades/kg/24 h de FEIBA, porque puede asociarse a riesgo de tromboembolismo venoso o coagulación intravascular diseminada ^23^^,^^24^, además que no debe administrarse junto a ácido tranexámico, por asociarse con eventos tromboembólicos ^23^^,^^24^. En el segundo grupo, la terapia de primera línea consiste en una combinación de esteroides solos (dosis de 1mg/kg/día) o asociados a ciclofosfamida a bajas dosis (1-2 mg/kg/día), entre 3 a 5 semanas ^25^. Ante el fracaso del tratamiento de primera línea, la alternativa de elección es el rituximab ^25^. La recomendación habitual consiste en la administración de dosis semanales de 375 mg/semana durante 4 semanas ^26^^,^^27^.

La corrección parcial al realizar la prueba de mezcla o corrección con plasma normal también se ha mencionado en reportes anteriores ^15^. Pudo deberse a aspectos analíticos como preanalíticos que escapan del motivo de este reporte ^27^. La anamnesis detallada, sumado a los hallazgos de laboratorio (reducción de la actividad del factor VIII, presencia de inhibidores anti-FVIII 0,6 Unidades Bethesda/ml) ^28^, y respuesta al tratamiento permitieron realizar el diagnóstico final.

Entre las limitaciones de este reporte se encuentra el haber administrado enoxaparina a un paciente con un trastorno adquirido de la coagulación, por hallazgo de trombosis venosa profunda en miembro inferior izquierdo. Esta decisión agravó el cuadro de la paciente. Al respecto se señala que este hallazgo pudiera haber sido interpretado de manera errónea (la especificidad de la ecografía Doppler de color para el diagnóstico es dependiente del operador, la literatura señala valores entre 62 a 83%) ^29^; y la segunda es que sí existió un cuadro trombótico. Sin embargo, en esta última situación se debió considerar las manifestaciones hemorrágicas que ya tenía la paciente, por lo que se debió referir oportunamente para la realización de exámenes complementarios.

En conclusión, el reporte brinda información sobre el diagnóstico y tratamiento de un caso de HAA en el posparto. Es importante sospechar de esta patología en una paciente puérpera con persistencia de sangrado por herida operatoria, equimosis extensa y sin antecedente de sangrados.
